# The Use of Tranexamic Acid in Breast Reduction and Abdominoplasty: A Review of a Multicenter Federated Electronic Health Record Database

**DOI:** 10.1093/asjof/ojae077

**Published:** 2024-09-09

**Authors:** Theodore E Habarth-Morales, Emily Isch, Alexander P Zavitsanos, Wesley M Wride, Harrison D Davis, Arturo J Rios-Diaz, Robyn B Broach, John P Fischer, Joseph M Serletti, Said C Azoury, Matthew Jenkins

## Abstract

**Background:**

Tranexamic acid (TXA), a fibrinolysis inhibitor, is widely used in various surgical fields to minimize blood loss. However, its efficacy and safety in plastic surgery, especially in reduction mammaplasty and abdominoplasty, remain underexplored. This study investigates the utility of intravenous (IV) TXA in these procedures, focusing on reducing postoperative complications and evaluating its safety in the context of venous thromboembolism (VTE).

**Objectives:**

To evaluate the efficacy and safety of TXA in reduction mammaplasty and abdominoplasty.

**Methods:**

Using data from the TriNetX LLC (Cambridge, MA) National Health Research Network database, this retrospective study compared adult patients undergoing reduction mammaplasty or abdominoplasty who received intraoperative IV TXA against those who did not. Primary outcomes included postoperative seroma and hematoma incidences, whereas secondary outcomes assessed the necessity for procedural drainage and the occurrence of VTE within 1-year postsurgery.

**Results:**

No significant differences in the rates or risks of hematoma, seroma, or the need for procedural drainage between patients administered IV TXA and those who were not, for both reduction mammaplasty and abdominoplasty. Additionally, IV TXA did not increase the risk of VTE in either patient group.

**Conclusions:**

IV TXA application in reduction mammaplasty and abdominoplasty does not provide added benefits in reducing postoperative complications such as seroma, hematoma, or the necessity for procedural drainage. Furthermore, it does not alter the risk of thromboembolic events. These findings highlight the need for further research, particularly through randomized control trials, to understand TXA's efficacy in plastic surgery.

**Level of Evidence: 3 (Therapeutic):**

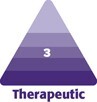

In the past decade, the employment of tranexamic acid (TXA) across various surgical specialties to mitigate blood loss and bleeding complications in elective and nonelective settings has gained popularity.^1,2^ Its effectiveness in plastic surgery has been demonstrated by diminishing bleeding complications in craniofacial, burn, and some cosmetic procedures.^3^ However, these procedures represent only a fraction of the annual plastic and reconstructive surgeries in the United States, suggesting that a broader spectrum of patients could benefit from its use.^4^ Therefore, it is essential to further investigate its safety profile and efficacy in common plastic surgeries, and explore the potential for wider application.

Data are scarce on the efficacy of TXA in 2 prevalent plastic procedures, namely reduction mammaplasty and abdominoplasty. These gaps become especially relevant to plastic surgeons as abdominoplasty remains the second most common abdominal body contouring surgery behind liposuction, whereas breast reductions saw the highest year-over-year increase of any plastic surgery procedure postpandemic.^4^ Common complications of these procedures, seroma, and hematoma could potentially be decreased by the administration of TXA. Despite the known adverse impact of these complications on patient-reported outcomes,^5-7^ only a few studies have attempted to address this question, yielding conflicting results.^8-11^ Additionally, there exists great heterogeneity among these studies in terms of routes of administration as TXA is commonly used topically, intravenously (IV), or both. Furthermore, the safety of TXA, particularly concerning the risk of venous thromboembolism (VTE) in the postdischarge period remains uncertain. These gaps in knowledge in plastic surgery may be preventing broader TXA adoption into clinical practice, and therefore, a study targeting this patient population is warranted.

Bearing this in mind, we sought to evaluate the safety profile and efficacy of TXA in reducing seroma, hematoma, and blood transfusion in patients undergoing reduction mammaplasty and abdominoplasty at a population level. We hypothesized that the benefits of IV TXA in reducing bleeding complications and seromas extend to this patient population when examined on a large scale.

## METHODS

### Study Design and Patient Population

This was a retrospective cohort study using the TriNetX LLC (Cambridge, MA) National Health Research Network database. All adult patients undergoing either abdominoplasty or reduction mammaplasty, as defined by procedure codes from the *International Classification of Diseases-Procedure Coding System-9th and 10th Revisions* and *Current Procedural Terminology* (ICD 9 and 10-PCSs and CPT, Supplemental Table 1) within the last 20 years were included. Patients were only included if their encounter included a code for “Encounter for Cosmetic Surgery—Z41.1.” Dates of inclusion were dependent on the dates of enrollment of a center's electronic health record (EHR) in the database and were thus variable between reduction mammaplasties and abdominoplasties, although all index surgeries occurred within the last 20 years. The database was queried on March 11, 2024 and analyses were conducted between March and April 2024.

### Data Source

The TriNetX LLC National Health Research Network database is a regularly updated repository of de-identified healthcare information compiled from the EHRs of over 100 million patients across 70 healthcare organizations. It includes EHR data such as diagnoses, procedures, medications, laboratory values, and genomic data, which are abstracted from billing and procedure codes within the CPT and ICD. In addition, other data are abstracted from unstructured data through natural language processing which is then coded into variables. Natural language processing is a computational technique in which algorithms and artificial intelligence are used to convert unstructured data (ie, patient notes) into data variables. TriNetX, LLC ensures compliance with the Health Insurance Portability and Accountability Act (HIPAA) and adhere to de-identification standards outlined in the HIPAA Privacy Rule. As this study exclusively utilized de-identified patient records and did not involve the collection, use, or transmission of individually identifiable data, Institutional Review Board approval was deemed unnecessary.

### Data Elements

Demographics data extracted included age at the time of procedure, ethnicity, race, and BMI. Relevant diagnoses, such as primary hypertension, diabetes mellitus Type 2, chronic coagulopathy, and active tobacco use, were also ascertained. For reduction mammaplasties, information such as concurrent procedures (abdominoplasty and liposuction) was obtained. For abdominoplasties, concurrent liposuction was also noted. Perioperative deep vein thrombosis (DVT) prophylaxis was also ascertained for both cohorts. Patients from both cohorts of abdominoplasties and breast reductions were then stratified by the use of intraoperative IV TXA administration on the day of surgery. All patients analyzed had a documented follow-up for at least 1 year after the index procedure.

### Outcome Measures

The primary endpoint of the study was the incidence of postoperative seroma and hematoma. Secondary outcomes included the need for postoperative procedural drainage of seroma/hematoma, the incidence of pulmonary embolism or DVT (grouped as VTE), seizures, as well as infectious complications. Transfusion of blood products within 72 h was also extracted.

### Statistical Analysis

Descriptive statistics (counts and proportions) were used to report demographic, clinical, and hospital-level characteristics. Continuous variables were reported as their median value with the interquartile range. Raw univariate comparisons of covariates and primary and secondary outcomes were performed followed by subsequent propensity score matching based on variables identified a priori as potential confounders. The variables selected were age, sex, race, ethnicity, medical comorbidities (hypertension, diabetes, previously diagnosed VTE or coagulopathy, and history of tobacco use), and operative variables such as concurrent liposuction and intraoperative hemorrhagic complications. After matching both sets of cohorts, covariates were well balanced. Multivariate logistic regression was subsequently performed on matched cohorts to evaluate the adjusted risk of hematoma, seroma, procedural drainage, and VTE with the use of TXA during index surgery. Patients with inadequate follow-up or missing data were excluded from multivariate analyses and totaled 2.7%. Per data use agreements, cells with counts of <10 instances in tables were reported as 10. All analyses were performed within the TriNetX web-based analytics interface and significance was set at *P* < .005.

## RESULTS

### Reduction Mammaplasty

A total of 62,639 patients underwent reduction mammaplasty during the study period (2004-2024). The median age was 41.8 years (standard deviation [SD] 15.9), 54.6% were White, 6.6% were Hispanic–Latino, and the median BMI of the cohort was 31.6 (SD 6.0). The baseline medical history of the population demonstrated 13,244 (21.1%) patients with hypertension, 4512 (7.2%) with Type 2 diabetes, 1644 (2.6%) had a history of tobacco use, 727 (1.1%) had a history of VTE, 508 (0.8%) had previously diagnosed coagulopathy, and 10,555 (16.9%) had postoperative thromboprophylaxis (Table 1). Additionally, 1958 (3.1%) had concurrent liposuction.

**Table 1.** Demographic and Operative Characteristics of Patients Undergoing Reduction Mammaplasty by the Use of Intravenous Tranexamic Acid

*n*		Before matching								After matching					
		Total	TXA		No TXA				Total	TXA		No TXA			
		62,639	1708		58,899		*P*-value	Standardized difference	3414	1707		1707		*P*-value	Standardized difference
		*n*/mean	*n*/mean	(%/SD)	*n*/mean	(%/SD)				*n*/mean	(%/SD)	*n*/mean	(%/SD)		
Age, years		41.8	38.4	(15.5)	41.9	(16.1)	<.001	0.223	38.5	38.4	15.5	38.5	15.2	.833	0.007
Race	White	34,226	973	(57.0)	33,253	(56.5)	<.001	0.039	1926	972	(56.9)	954	(55.9)	.534	0.021
	African American	13,776	428	(25.1)	13,348	(22.7)			880	428	(25.1)	452	(26.5)		
	Asian	630	32	(1.9)	598	(1.0)			54	32	(1.9)	22	(1.3)		
	Other	2935	114	(6.7)	2821	(4.8)			217	114	(6.7)	103	(6.0)		
Ethnicity	Hispanic–Latino	4175	192	(11.2)	3983	(6.8)	<.001	0.146	391	191	(11.2)	200	(11.7)	.629	0.017
BMI		31.6	31.3	(6.2)	31.6	(6.0)	.186	0.046	31.6	31.3	6.2	31.8	6.3	.129	0.074
Hypertension		13,249	366	(21.4)	12,883	(21.9)	.176	0.034	739	366	(21.4)	373	(21.9)	.771	0.010
Type 2 diabetes		4512	114	(6.7)	4398	(7.5)	.089	0.043	219	114	(6.7)	105	(6.2)	.53	0.022
History of coagulopathy		508	22	(1.3)	486	(0.8)	.062	0.041	39	22	(1.3)	17	(1.0)	.421	0.028
History of VTE		727	18	(1.1)	709	(1.2)	.459	0.019	33	18	(1.1)	15	(0.9)	.600	0.018
Postoperative VTE prophylaxis		10,555	301	(17.6)	10,254	(17.4)	.566	0.014	596	301	(17.6)	295	(17.3)	.787	0.009
History of tobacco use		1644	46	(2.7)	1598	(2.7)	.186	0.046	93	46	(2.7)	47	(2.8)	.915	0.005
Concurrent liposuction		1958	66	(3.9)	1892	(3.2)	.248	0.027	138	66	(3.9)	72	(4.2)	.602	0.018

SD, standard deviation; TXA, tranexamic acid; VTE, venous thromboembolism.

Of all patients undergoing reduction mammaplasties, 1708 (2.7%) were administered IV TXA on the day of their procedure. Patients in the TXA intervention group were more likely to be younger, White, and Hispanic–Latino, although they had no differences in terms of comorbidities compared with those without TXA administration. Subsequently, 1707 pairs of patients were matched from both cohorts. The matched cohorts were well balanced. Seromas occurred in 19 (1.1%) patients administered IV TXA compared with 12 (0.7%) in the control group (*P* = .157). Hematomas occurred in 24 (1.4) intervention patients vs 23 (1.3%) in the control group (*P* = .531). There was no difference in the rate of procedural drainage events postoperatively (1.6% vs 2.0%, *P* = .556) or blood transfusions (0.6% vs 0.6%, *P* = 1.000). Additionally, there was no difference in the number of postoperative VTE events (10% vs 12%, *P* = .880) or seizures (0.6% vs 0.5%, *P* = .411; Table 2).

**Table 2.** Univariate and Adjusted Risk of Outcomes in Patients Undergoing Reduction Mammaplasty With or Without Intravenous Tranexamic Acid

	TXA		No TXA				
	*n* = 1707		*n* = 1707				
	*n*	(%)	*n*	(%)	Odds ratio (ref. no TXA)	(95% CI)	*P*-value
Seroma	19	(1.1)	12	(0.7)	1.590	(0.769-3.286)	.207
Hematoma	24	(1.4)	23	(1.3)	1.044	(0.587-1.857)	.883
Infection	58	(3.4)	60	(3.5)	0.965	(0.669-1.394)	.851
Procedural drainage	27	(1.6)	34	(2.0)	0.791	(0.475-1.317)	.366
Blood transfusion	10	(0.6)	10	(0.6)	1.000	(0.415-2.409)	1.000
VTE	10	(0.6)	12	(0.7)	0.832	(0.359-1.932)	.669

TXA, tranexamic acid; VTE, venous thromboembolism.

After controlling for relevant confounders, there was no difference observed in the risk of postoperative seroma (odds ratio [OR]: 1.590, 95% CI: 0.769-3.286, *P* = .207), hematoma (OR: 1.044, 95% CI: 0.587-1.857, *P* = .883), or need for procedural drainage (OR: 0.791, 95% CI: 0.475-1.317, *P* = .366) or blood transfusion (OR: 1.000, 95% CI: 0.415-2.409, *P* = 1.000). Finally, there was no difference in the risk of VTE with the administration of IV TXA (OR: 0.832, 95% CI: 0.359-1.932, *P* = .669).

### Abdominoplasty

A total of 13,394 patients underwent abdominoplasty during the study period (2004-2024). The mean age was 45.2 years (SD 11.4), 65.9% were White, 10.4% were Hispanic–Latino, and the median BMI of the cohort was 29.2 (SD 5.49). The medical baseline of this population was 3849 (28.7%) patients with hypertension, 1941 (14.5%) with Type 2 diabetes, 357 (2.7%) with a history of tobacco use, 279 (2.1%) with a documented history of VTE, 269 (2%) had previously diagnosed coagulopathy, and 3769 (28.1%) had postoperative thromboprophylaxis (Table 3). Additionally, 4989 (37.2%) had concurrent liposuction.

**Table 3.** Demographic and Operative Characteristics of Patients Undergoing Abdominoplasty by the Use of Intravenous Tranexamic Acid

*n*		Before matching							After matching						
		Total	TXA		No TXA				Total	TXA		No TXA			
		13,394	473		12,921		*P*-value	Standardized difference	930	465		465		*P*-value	Standardized difference
			*n*/mean	(%/SD)	*n*/mean	(%/SD)				*n*/mean	(%/SD)	*n*/mean	(%/SD)		
Age, years			47	(11.3)	45.3	(11.4)	.001	0.157		47.2	(11.7)	47.1	(11.3)	.982	0.001
Female			407	(86)	11,529	(89.3)	.027	0.098		399	(85.8)	409	(88.0)	.331	0.064
Race	White		273		8555		<.001	0.176		272		286		.349	0.061
	African American		96		1673					90		85			
	Asian		11		183					10		10			
	Other		93		1118					16		15			
Ethnicity	Hispanic–Latino		60	(12.7)	1339	(10.4)	.106	0.073		59	(12.7)	65	(14.0)	.563	0.038
BMI			29.2	(5.3)	29.2	(5.5)	.839	0.011		29.1	(5.3)	29.8	(5.4)	.076	0.132
Hypertension			155	(32.8)	3694	(28.6)	.049	0.090		153	(32.9)	134	(28.8)	.177	0.089
Type 2 diabetes			70	(14.8)	1871	(14.5)	.850	0.009		73	(15.7)	59	(12.7)	.188	0.086
History of coagulopathy			23	(4.9)	246	(1.9)	<.001	0.164		21	(4.5)	18	(3.9)	.624	0.032
History of VTE			10	(2.1)	269	(2.1)	.963	0.002		10	(2.2)	10	(2.2)	1.000	<0.001
Postoperative VTE prophylaxis			128	(27.1)	3641	(28.2)	.591	0.025		128	(27.5)	123	(26.5)	.712	0.024
History of tobacco use			18	(3.8)	339	(2.6)	.118	0.067		17	(3.7)	17	(3.7)	1.000	<0.001
Concurrent liposuction			136	(28.8)	4011	(31.1)	.287	0.050		134	(28.8)	127	(27.3)	.609	0.034

SD, standard deviation; TXA, tranexamic acid; VTE, venous thromboembolism.

Of all patients undergoing abdominoplasty, 473 (3.5%) were administered IV TXA on the day of their procedure. Patients in the TXA intervention group were more likely to be younger, White, and Hispanic–Latino, although they had no differences in terms of comorbidities compared with those without TXA administration. Subsequently, 465 pairs of patients were matched from both cohorts. The matched cohorts were well balanced. Seromas occurred in 19 (4.1%) patients administered TXA compared with 15 (3.2%) in the control group (*P* = .782). Hematomas occurred in 14 (3%) patients vs 10 (2.2%) in the control group (*P* = .708). There was no significant difference in the rate of procedural drainage events postoperatively (2.2% vs 2.2%, *P* = 1.000) or blood transfusions (2.2% vs 2.2%, *P* = 1.000). Additionally, there was no significant difference in the number of postoperative VTE events (2.2% vs 2.2%, *P* = 1.000) or seizures (0.9% vs 0.9%, *P* = 1.000).

After controlling for relevant confounders, there was no difference observed in the risk of postoperative seroma (OR: 0.782, 95% CI: 0.393-1.559, *P* = .485), hematoma (OR: 0.708, 95% CI: 0.311-1.611, *P* = .408), or need for procedural drainage (OR: 1.00, 95% CI: 0.412-2.426, *P* = 1.000). The risk for blood transfusion remained unchanged (OR: 1.00, 95% CI: 0.412-2.426, *P* = 1.000). Finally, there was no significant difference in the risk of VTE with the administration of IV TXA (OR: 1.00, 95% CI: 0.412-2.426, *P* = 1.000; Table 4).

**Table 4.** Univariate and Adjusted Risk of Outcomes in Patients Undergoing Abdominoplasty With or Without Intravenous Tranexamic Acid

	TXA		No TXA		Odds ratio (ref. no TXA)	(95% CI)	*P*-value
	*n* = 465		*n* = 465				
	*n*	(%)	*n*	(%)			
Seroma	19	(4.1)	15	(3.2)	0.782	(0.393-1.559)	.485
Hematoma	14	(3.0)	10	(2.2)	0.708	(0.311-1.611)	.408
Infection	23	(4.9)	30	(6.5)	1.325	(0.758-2.318)	.322
Procedural drainage	<10^a^	(2.2)	<10^a^	(2.2)	1.000	(0.412-2.426)	1.000
Blood transfusion	<10^a^	(2.2)	<10^a^	(2.2)	1.000	(0.412-2.426)	1.000
VTE	<10^a^	(2.2)	<10^a^	(2.2)	1.000	(0.412-2.426)	1.000

TXA, tranexamic acid; VTE, venous thromboembolism. ^a^Cells with <10 instances are reported as <10 per data use agreement for privacy reasons.

## DISCUSSION

TXA is a synthetic lysine derivative that prevents the conversion of plasminogen to plasmin thereby preventing fibrinolysis. Unsurprisingly, since its FDA approval in 1986, TXA has been increasingly used to stop and/or prevent bleeding and has shown a great deal of efficacy in doing so in specific situations, such as trauma, postpartum hemorrhage, and a variety of other surgical procedures.^12-15^ Although the use of IV TXA in orthopedic, cardiac, and obstetric surgeries has been common, the use of TXA in the selective plastic surgery procedures is relatively new. Thus, there exists a reasonable gap in the literature regarding both the efficacy and safety of its use in common plastic surgery procedures. In a retrospective review of 3414 abdominoplasties and 27,402 reduction mammaplasties in an aggregate federated EHR database from 70 health systems, there was no difference in the rate or risk of hematoma or seroma formation, need for procedural seroma/hematoma drainage, or postoperative VTE within 1-year postoperative with use of IV TXA.

TXA has been used extensively in other surgical subspecialties for the control of blood loss.^14,15^ The use of TXA in plastic surgery has vastly been limited to craniofacial surgery until recently, and until 2018, only 3 relevant articles had been published in the plastic surgery literature.^16-18^ At the time of composing this article, 87 articles exist on the topic of TXA in plastic surgery. The use of TXA has expanded in recent years to rhinoplasty and other aesthetic facial procedures, such as facelifts, and in a recent study, Brown et al found nearly 1 in 6 plastic surgeons are using TXA in aesthetic surgery.^19-24^ However, in reduction mammaplasty and abdominoplasty, 2 of the most common body contouring procedures performed nationwide, there is conflicting literature among the few studies in existence. In a retrospective chart review of 191 patients, Om et al utilized a cohort of 74 patients undergoing reduction mammaplasty with 1 g administration of intraoperative IV TXA to evaluate bleeding outcomes, such as hematoma and seroma rates. This study found a 14.82% higher rate of hematoma formation in the TXA arm vs the control arm and found no difference in terms of seroma.^11^ In their larger study, Weissler et al at the Mayo Clinic of 385 patients undergoing reduction mammaplasty, 66.8% of whom received IV TXA, found no decrease in the risk of hematoma and seroma formation.^10^ Similarly, our study of 4222 matched pairs found no difference in the rate of seroma/hematoma formation, use of blood products, or need for procedural drainage. Other studies, including 1 randomized control trial, have also been performed evaluating the efficacy of topically administered TXA in reduction mammaplasty. These studies have largely found decreased bleeding complications, indicating that perhaps topical administration may be a more efficacious alternative to IV administration.^9,25^ Our study, importantly, only evaluated parenteral use, and other routes of administration should be studied individually to provide evidence for or against its use in these other cases.

In addition to breast reductions, body contouring has also been targeted as a potential procedure whereby TXA could be used as an adjunctive therapy. Intravenous and local TXA was associated with lower blood loss in large-volume liposuction,^26^ and IV TXA has been found to decrease blood loss in general liposuction procedures.^27^ However, the evaluation of TXA strictly in abdominoplasties is worth examining as it is another common modality for body contouring with higher rates of postoperative seroma and hematoma. Although the literature on this topic is sparse, 1 retrospective review found no difference in the rate of bleeding complications among patients undergoing “trunk aesthetic surgery” who were administered TXA.^28^ Another study of 288 patients undergoing panniculectomy found no difference in rates or risk of hematoma or seroma formation, or duration of drain placement with the administration of topical TXA.^8^ It is clear that supported evidence-based techniques, such as meticulous intraoperative hemostasis, the use of drains, and obliteration of potential dead space, remain the most effective way to reduce these complications and further randomized control trials that are powered for evaluating differences in these complications are warranted.

Finally, the safety of TXA is well-studied in the other surgical literature.^29-32^ Of particular concern is the theoretical risk of VTE following the administration of TXA. Most studies have supported the conclusion that there is no increased risk of VTE conferred with its use,^33,34^ although a few other publications have suggested an unclear safety margin.^35,36^ In plastic surgery, the extant literature has largely supported the safety of TXA. A systematic review of 23 publications found there to be no association between TXA use and postoperative thrombosis/thromboembolism.^37^ Multiple meta-analyses found that TXA administration had no significant association with an increased likelihood of thrombosis in breast surgery.^38,39^ However, there have been no studies published examining thrombotic events specifically in reduction mammaplasties, although our findings seem to corroborate the conclusions of the aforementioned studies within that cohort. A 427-participant randomized control trial in liposculpture and abdominoplasty demonstrated no association between presurgical and postsurgical TXA use in thrombotic events,^40^ again supporting the framing of our results. Another important adverse effect of TXA that has been cited in the literature regards a rarely described increase in neurological complications such as seizures.^41,42^ Our results corroborate other evidence in the plastic surgery literature that there is no associated increase in the risk of seizures with its use.^43^

This study is not without its limitations, primarily those inherent to a retrospective database review. The power offered by a federated EHR database, such as TriNetX, naturally compromises granular information which could confound results such as the number of drains used, operative duration, time to drain removal, and volume of tissue resection, among others. Although the ability to follow-up with patients and diagnosis codes both inpatient and outpatient offers considerable strength to the study, there remains a chance that some patients did experience complications at hospitals or clinics which were not part of the database network. Retrospective studies are naturally prone to selection bias particularly because of diagnostic and procedure coding, although the new Natural Language Processing initiative by TriNetX aims to reduce selection bias by mining unstructured data within the health record. Another limitation of our study is the unavailability of dosage information and there presumably exists some heterogeneity of formulation administered in our cohort. Lastly, some selection bias may exist from the healthcare systems participating in TriNetX as most institutional participants are primarily larger academic centers.

## CONCLUSIONS

Although TXA use in a variety of surgical procedures has been commonplace for many years, the field of plastic surgery has only recently begun exploring its use. The present study found no additional benefit to use TXA in reduction mammaplasty or abdominoplasty. However, more importantly, TXA did not increase the risk of thromboembolic complications after its use. These findings highlight the importance of continued research into TXA use in plastic surgery through randomized control trials powered to detect differences in these complications.

## Supplemental Material

This article contains supplemental material located online at https://doi.org/10.1093/asjof/ojae077.

## Supplementary Material

ojae077_Supplementary_Data

## References

[1] Olaleye AA , Adebayo JA, Eze JN, et al Efficacy of tranexamic acid in reducing myomectomy-associated blood loss among patients with uterine myomas at Federal Teaching Hospital Abakaliki: a randomized control trial. Int J Reprod Med. 2024;2024:2794052. doi: 10.1155/2024/2794052 38283394 PMC10810692

[2] Gibbs VN , Champaneria R, Palmer A, et al Pharmacological interventions for the prevention of bleeding in people undergoing elective hip or knee surgery: a systematic review and network meta-analysis. Cochrane Database Syst Rev. 2019;1(1):CD013295. doi: 10.1002/14651858.CD013295 PMC10790339 38226724

[3] Brown S , Brown T, Rohrich RJ. Clinical applications of tranexamic acid (TXA) in plastic and reconstructive surgery. Plast Reconstr Surg. 2024. doi: 10.1097/PRS.0000000000011288. [Epub ahead of print] 38196097

[4] American Society of Plastic Surgeons . American Society of Plastic Surgeons Reveals 2022s Most Sought-After Procedures. ASPS Media Relations; 2023.

[5] Mohammad JA , Warnke PH, Stavraky W. Ultrasound in the diagnosis and management of fluid collection complications following abdominoplasty. Ann Plast Surg. 1998;41(5):498–502. doi: 10.1097/00000637-199811000-00008 9827952

[6] Wampler AT , Powelson IA, Homa K, Freed GL. BREAST-Q outcomes before and after bilateral reduction mammaplasty. Plast Reconstr Surg. 2021;147(3):382e–390e. doi: 10.1097/PRS.0000000000007605 33620922

[7] Greco R , Noone B. Evidence-based medicine: reduction mammaplasty. Plast Reconstr Surg. 2017;139(1):230e–239e. doi: 10.1097/PRS.0000000000002856 28027257

[8] Weissler JM , Kuruoglu D, Salinas C, et al Defining the role for topically administered tranexamic acid in panniculectomy surgery. Aesthet Surg J Open Forum. 2022;4:ojac033. doi: 10.1093/asjof/ojac033 35692487 PMC9174740

[9] Ausen K , Fossmark R, Spigset O, Pleym H. Randomized clinical trial of topical tranexamic acid after reduction mammoplasty. Br J Surg. 2015;102(11):1348–1353. doi: 10.1002/bjs.9878 26349843 PMC4600231

[10] Weissler JM , Kuruoglu D, Antezana L, et al Efficacy of tranexamic acid in reducing seroma and hematoma formation following reduction mammaplasty. Aesthet Surg J. 2022;42(6):616–625. doi: 10.1093/asj/sjab399 35029651

[11] Om A , Marxen T, Kebede S, Losken A. The usage of intravenous tranexamic acid in reduction mammaplasty safely reduces hematoma rates. Ann Plast Surg. 2023;90(6S):S371–S374. doi: 10.1097/SAP.0000000000003296 36729851 PMC10578999

[12] CRASH-2 Trial Collaborators, Shakur H, Roberts I, et al Effects of tranexamic acid on death, vascular occlusive events, and blood transfusion in trauma patients with significant haemorrhage (CRASH-2): a randomised, placebo-controlled trial. Lancet. 2010;376(9734):23–32. doi: 10.1016/S0140-6736(10)60835-5 20554319

[13] Ducloy-Bouthors A-S , Jude B, Duhamel A, et al High-dose tranexamic acid reduces blood loss in postpartum haemorrhage. Crit Care. 2011;15(2):R117. doi: 10.1186/cc10143 21496253 PMC3219400

[14] Gandhi R , Evans HMK, Mahomed SR, Mahomed NN. Tranexamic acid and the reduction of blood loss in total knee and hip arthroplasty: a meta-analysis. BMC Res Notes. 2013;6(1):184. doi: 10.1186/1756-0500-6-184 23651507 PMC3655041

[15] Devereaux PJ , Marcucci M, Painter TW, et al Tranexamic acid in patients undergoing noncardiac surgery. N Engl J Med. 2022;386(21):1986–1997. doi: 10.1056/NEJMoa2201171 35363452

[16] Engel M , Bodem JP, Busch CJ, et al The value of tranexamic acid during fronto-orbital advancement in isolated metopic craniosynostosis. J Craniomaxillofac Surg. 2015;43(7):1239–1243. doi: 10.1016/j.jcms.2015.05.004 26116972

[17] Goobie SM , Meier PM, Pereira LM, et al Efficacy of tranexamic acid in pediatric craniosynostosis surgery: a double-blind, placebo-controlled trial. Anesthesiology. 2011;114(4):862–871. doi: 10.1097/ALN.0b013e318210fd8f 21364458

[18] Nayak LM , Linkov G. The role of tranexamic acid in plastic surgery. Plast Reconstr Surg. 2018;142(3):423e. doi: 10.1097/PRS.0000000000004669 29965923

[19] Albazee E . Letter on tranexamic acid in patients undergoing rhinoplasty: an updated systematic review and meta-analysis of randomized controlled trials. Aesthet Plast Surg. 2024;48(11):2086–2087. doi: 10.1007/s00266-024-03868-8 38332054

[20] Gutierrez RWH , Gobbo HR, da Heringer LFL. Tranexamic acid in patients undergoing rhinoplasty: an updated systematic review and meta-analysis of randomized controlled trials. Aesthet Plast Surg. 2023;48(11):2076–2085. doi: 10.1007/s00266-023-03768-3 38097691

[21] Ricca F , Spiegel JH. What is the role of tranexamic acid in septorhinoplasty? Laryngoscope. 2024;134(1):3–4. doi: 10.1002/lary.30880 37431883

[22] Wu B , Chen S, Sun K, Xu X. Complications associated with rhinoplasty: an umbrella review of meta-analyses. Aesthet Plast Surg. 2022;46(2):805–817. doi: 10.1007/s00266-021-02612-w 34590168

[23] Al-Hashimi M , Kaur P, Charles W, et al A systematic review of the efficacy and safety of tranexamic acid in facelift surgery. Aesthet Surg J. 2023;43(11):1211–1218. doi: 10.1093/asj/sjad213 37402636

[24] Brown S , Brown T, Taub P, Rohrich R. The role of tranexamic acid in plastic and reconstructive surgery: a national perspective. Plast Reconstr Surg Glob Open. 2021;9(10S):21. doi: 10.1097/01.GOX.0000799192.39058.b8

[25] Lonie S , Abesamis GM, Law J, et al Topical tranexamic acid in primary breast augmentation surgery: short- and long-term outcomes. Aesthet Surg J. 2023;44(1):NP23–NP27. doi: 10.1093/asj/sjad219 37427875

[26] Minawi HME , Kadry HM, El-Essawy NM, et al The effect of tranexamic acid on blood loss in liposuction: a randomized controlled study. Eur J Plast Surg. 2023;46(2):227–237. doi: 10.1007/s00238-022-01995-6 36311870 PMC9589853

[27] Reinhardt ME , Mutyala S, Gerald M, et al The critical blood-sparing effect of tranexamic acid (TXA) in liposuction: a systematic review and meta-analysis. JPRAS Open. 2024;40:48–58. doi: 10.1016/j.jpra.2023.01.002 38425698 PMC10904189

[28] Neel OF , AlKhashan R, AlFadhel EA, et al Use of tranexamic acid in aesthetic surgery: a retrospective comparative study of outcomes and complications. Plast Reconstr Surg Glob Open. 2023;11(9):e5229. doi: 10.1097/GOX.0000000000005229 37662475 PMC10473321

[29] Dai Z , Chu H, Wang S, Liang Y. The effect of tranexamic acid to reduce blood loss and transfusion on off-pump coronary artery bypass surgery: a systematic review and cumulative meta-analysis. J Clin Anesth. 2018;44:23–31. doi: 10.1016/j.jclinane.2017.10.004 29107853

[30] Nguyen A , Brown NJ, Gendreau J, et al The association of thromboembolic complications and the use of tranexamic acid during resection of intracranial meningiomas: systematic review and meta-analysis of randomized controlled trials. J Neurosurg. 2023;140(1):1–11. doi: 10.3171/2023.7.JNS23849 37856372

[31] Hess MC , Andrews NA, Crowley B, et al Intravenous tranexamic acid decreases intraoperative transfusion requirements and does not increase incidence of symptomatic venous thromboembolic events in musculoskeletal sarcoma surgery. Surg Oncol. 2023;50:101989. doi: 10.1016/j.suronc.2023.101989 37717375

[32] Brown NJ , Pennington Z, Himstead AS, et al Safety and efficacy of high-dose tranexamic acid in spine surgery: a retrospective single-institution series. World Neurosurg. 2023;177:e18–e25. doi: 10.1016/j.wneu.2023.04.058 37141940

[33] Taeuber I , Weibel S, Herrmann E, et al Association of intravenous tranexamic acid with thromboembolic events and mortality: a systematic review, meta-analysis, and meta-regression. JAMA Surg. 2021;156(6):e210884. doi: 10.1001/jamasurg.2021.0884 33851983 PMC8047805

[34] Ker K , Edwards P, Perel P, et al Effect of tranexamic acid on surgical bleeding: systematic review and cumulative meta-analysis. BMJ. 2012;344:e3054. doi: 10.1136/bmj.e3054 22611164 PMC3356857

[35] Karl V , Thorn S, Mathes T, et al Association of tranexamic acid administration with mortality and thromboembolic events in patients with traumatic injury: a systematic review and meta-analysis. JAMA Netw Open. 2022;5(3):e220625. doi: 10.1001/jamanetworkopen.2022.0625 35230436 PMC8889461

[36] HALT-IT Trial Collaborators . Effects of a high-dose 24-h infusion of tranexamic acid on death and thromboembolic events in patients with acute gastrointestinal bleeding (HALT-IT): an international randomised, double-blind, placebo-controlled trial. Lancet. 2020;395(10241):1927–1936. doi: 10.1016/S0140-6736(20)30848-5 32563378 PMC7306161

[37] Elena Scarafoni E . A systematic review of tranexamic acid in plastic surgery: what's new? Plast Reconstr Surg Glob Open. 2021;9(3):e3172. doi: 10.1097/GOX.0000000000003172 33907653 PMC8062149

[38] Liechti R , van de Wall BJM, Hug U, et al Tranexamic acid use in breast surgery: a systematic review and meta-analysis. Plast Reconstr Surg. 2023;151(5):949–957. doi: 10.1097/PRS.0000000000010071 36729428

[39] Calpin GG , McAnena PF, Davey MG, et al The role of tranexamic acid in reducing post-operative bleeding and seroma formation in breast surgery: a meta-analysis. Surgeon. 2023;21(4):e183–e194. doi: 10.1016/j.surge.2022.11.005 36572609

[40] Bayter-Marín JE , Hoyos A, Cárdenas-Camarena L, et al Effectiveness of tranexamic acid in the postoperative period in body contour surgery: randomized clinical trial. Plast Reconstr Surg Glob Open. 2023;11(11):e5403. doi: 10.1097/GOX.0000000000005403 38025645 PMC10653580

[41] Mergoum AM , Mergoum AS, Larson NJ, et al Tranexamic acid use in the surgical arena: a narrative review. J Surg Res. 2024;302:208–221. doi: 10.1016/j.jss.2024.07.042. [Epub ahead of print] 39106732

[42] Deshpande DV , McKinley WI, Benjamin AJ, Schreiber MA, Rowell SE. The association between tranexamic acid and seizures in moderate or severe traumatic brain injury. J Surg Res. 2024;301:359–364. doi: 10.1016/j.jss.2024.06.035. [Epub ahead of print] 39024715

[43] Zaussinger M , Kerschbaumer C, Schwartz B, Bachleitner K, Ehebruster G, Schmidt M. Influence of tranexamic acid in body contouring surgery: significant changes on complication rates after abdominoplasty. Aesthetic Plast Surg. 2024;48(15):2872–2878. doi: 10.1007/s00266-024-04094-y. [Epub ahead of print] 38750226

