# Supplementary material for: The Use of Tranexamic Acid in Breast Reduction and Abdominoplasty: A Review of a Multicenter Federated Electronic Health Record Database
Source: Aesthet Surg J Open Forum. 2024 Sep 9;6:ojae077. doi: 10.1093/asjof/ojae077 (PMC11487907; doi:10.1093/asjof/ojae077)
Supplement: ojae077_Supplementary_Data [file ojae077_supplementary_data.docx]

SUPPLEMENTAL DIGITAL CONTENT

|  | CPT Code | ICD-10 Code | RxNorm |
| --- | --- | --- | --- |
| Abdominoplasty | 15830, 15847 |  |  |
| Reduction Mammaplasty | 19318 |  |  |
| Liposuction | 15877 |  |  |
| Procedural Drainage of Hematoma/Seroma/Fluid Collection | 10140 |  |  |
| Tranexamic Acid |  |  | 10691 |
| Direct Oral Anticoagulants |  |  | 1364430, 1114195 |
| Heparin |  |  | 5224, 67108, 67109 |
| Diabetes |  | E08.XX-E13.XX |  |
| Hypertension |  | I10.XX |  |
| History of Coagulopathy |  | Z86.2X |  |
| History of Venous Thromboembolism |  | Z86.71 |  |
| Tobacco Use |  | F17X, Z72.0X |  |
| Hematoma |  | L76.31, L76.32 |  |
| Seroma |  | L76.33, L76.34 |  |
| Venous Thromboembolism |  | I82.XX |  |

**Abbreviations**. CPT, current procedural terminology; ICD, international classification of diseases.
